# Grafting Tomato Scions on Root Knot Nematode (RKN)-Resistant Brinjal Rootstocks Complemented with Biocontrol Agents as an Integrated Nematode Management (INM) Strategy for the Development of RKN-Resistant Tomato

**DOI:** 10.3390/pathogens14121257

**Published:** 2025-12-08

**Authors:** Anil K. Poonia, Bhupendra Koul, Subhash Kajla, Meerambika Mishra, Muhammad Fazle Rabbee

**Affiliations:** 1 Department of Botany, School of Bioengineering and Biosciences, Lovely Professional University, Phagwara 144411, India; anil_poonia2005@yahoo.com; 2 Department of Molecular Biology and Biotechnology, College of Biotechnology, Chaudhary Charan Singh Haryana Agricultural University (CCSHAU), Hisar 125004, India; 3 Department of Biotechnology, School of Bioengineering and Biosciences, Lovely Professional University, Phagwara 144411, India; 4 Department of Botany, College of Basic Sciences and Humanities, Chaudhary Charan Singh Haryana Agricultural University (CCSHAU), Hisar 125004, India; ksubhash73@hotmail.com; 5 Department of Infectious Disease and Immunology, College of Veterinary Medicine, University of Florida, Gainesville, FL 32611, USA; meerambika.mishra@gmail.com; 6 Department of Biotechnology, Yeungnam University, Gyeongsan 38541, Republic of Korea

**Keywords:** root knot nematode, root knot index, *Meloidogyne incognita*, grafting, eggplant, biocontrol agents

## Abstract

Root knot nematodes (RKNs; *Meloidogyne* spp.) are among the biotic stressors that reduce growth and yield by 25–100% in solanaceous crops like tomato. The present study screened 35 eggplant accessions against RKNs (inoculum: 1 J2/g soil). Average root galls and egg masses per root ranged from 6.66 to 196.66 and from 4.66 to 192.66, respectively. Of the 35 accessions, BR3 was identified as resistant, exhibiting low galling index (6.66 galls/root) and egg mass count (4.66 egg masses/root), along with the highest total phenolic content (1515.92 μg/g). The shaft grafting of the susceptible tomato variety Hisar Arun (scion) onto resistant eggplant accession BR3 (rootstock) achieved a 90% success rate. Three biocontrol agents, namely, *Trichoderma viride*, *Paecilomyces lilacinus*, and *Pseudomonas fluorescence* were applied @2 g and 4 g per kg of soil to enhance resistance against RKNs in the grafted tomato plants (Hisar Arun variety). Among these, *P. lilacinus* at 4 g/kg soil reduced root galls, egg masses, and final nematode population by 84.65%, 95.7%, and 82.12%, respectively, compared with positive controls. Grafted plants treated with *P. lilacinus* at 4 g/kg soil also exhibited superior growth parameters relative to the control plants. Hence, the Integrated Nematode Management (INM) strategy developed in the present study can be used for delivering natural resistance against root knot nematodes in tomato plants and other solanaceous crops.

## 1. Introduction

Tomato (*Solanum lycopersicum* L.; *Solanaceae* family) ranks among the world’s most widely consumed vegetables. It features in diverse culinary preparations, consumed as soups, sauces, chutneys, and even eaten raw as salads. Moreover, it is an excellent source of lycopene (1–8 mg/100 g), minerals (K, Ca, Mg, P, Fe, Cu, Zn, and Mn), vitamins (C and K), and antioxidants. Tomato growth and yield suffer from various biotic and abiotic stresses [1,2,3]. Among biotic factors, root knot nematodes (*Meloidogyne* spp.) severely constrain vegetable production, causing 20–100% yield losses worldwide [2,4,5,6,7,8]. Four principal species, *M. incognita*, *M. arenaria*, *M. javanica*, and *M. hapla*, significantly impair plant growth and productivity. RKNs induce characteristic root galls (up to 1 inch in diameter) that restrict water and nutrient uptake, rendering infected plants less vigorous. Affected plants later yellow due to poor fertilizer response, exhibiting stunted growth, reduced fruit yield, wilting, and secondary pathogen susceptibility [7,8,9,10,11]. Chemical controls prove costly, temporary, and hazardous to human health, necessitating novel RKN management strategies.

Eggplant (*Solanum melongena* L.), commonly known as brinjal, is a native of the *Solanaceae* family and is a close relative of other important vegetable crops, like peppers, potatoes, and tomato. It is widely cultivated across the globe for its fruits, which are used as vegetables. Previously, the screenings of eggplants indicate that these plants may have some resistant genes against RKN infection [12,13,14,15]. One of the eggplant species, *Solanum torvum*, has been found to be extremely resistant against RKNs during physical screening and has been used as rootstock for grafting [16]. Moreover, a heat-stable gene, *SacMi*, has also been reported in wild eggplant *Solanum aculeatissimum*, which is similar to Mi genes and provides high resistance against RKNs [17]. This gene is expressed in response to infection by RKNs and confers a high level of resistance against the same. The silencing of the *SacMi* gene using RNAi changes the resistant plant into a susceptible plant, indicating a role of this gene in RKN resistance in eggplant. However, the screening of eggplant genotypes and cultivars for the presence of the *SacMi* gene has not been carried out so far. The primer for the screening of the *SacMi* gene has been developed [18] and can be used for the screening of eggplant germplasm for the presence of the *SacMi* gene. Many other molecular markers in related species are also available and can be used for molecular screening in eggplants. Molecular screening followed by marker-assisted breeding and genetic transformation can also serve as a tool to accelerate the introgression of RKN-resistant genes into susceptible crops [19,20,21]. However, the screening of plants for RKN resistance, genetic transformations, and breeding in vegetable crops are laborious, and introducing the RKN-resistant gene from the donor into host plants takes a long time [22].

Wild *Solanaceae* species harbor natural RKN resistance, making them suitable as donors [23,24,25]. Transferring resistance genes through breeding or transformation poses challenges. Resistant genes can be introduced into elite cultivars from a donor plant through breeding or genetic transformation to produce RKN-resistant cultivars; however, it is a key challenge for researchers to transfer a resistant gene through breeding or genetic transformation [21,26,27]. Therefore, there is a need to develop certain feasible techniques, like grafting, for the production of nematode-resistant tomato plants so as to avoid a yield penalty. To cope with the above-mentioned problems and to minimize all the losses and ill effects caused by nematodes, grafting could be one of the feasible and viable options in vegetable crops. It is a technique involving the deliberate joining of a rootstock and a scion of two living plants of compatible species or genera that ultimately grow as a single independent plant [28]. The rootstock is mostly a resistant rootstock which has an extensive root system for uptake of nutrients and resistance against soil-borne diseases, while the scion is a better-yielding or high-value variety. This eco-friendly approach reduces agrochemical reliance [29]. Tomato and eggplant belong to the same family, *Solanaceae*, and are thus compatible as scion and rootstock. Tomato plants produced through grafting using eggplant rootstocks not only showed resistance against soil-borne diseases like fungal wilt, bacterial wilt, and RKNs but also exhibited better growth and yield [23,28,30,31,32]. In this way, the disease resistance of the rootstock can be harnessed without transferring a foreign gene into the host plant.

The nematode can be controlled by chemicals in tomato, but it is a costly, toxic, and neither eco-friendly nor complete solution to the problem. Moreover, nowadays, nematicides and fumigants have been banned by several countries due to their adverse effects on human health, ozone, and the atmosphere [5]; hence, there is a need to develop an environmentally safe and cost-effective method for controlling RKNs [33,34]. Biocontrol agents such as fungi (*Trichoderma harzianum*, *Trichoderma viridae*, *Paecilomyces lilacinus*, etc.) and bacteria (*Pseudomonas fluorescens*, *B. amyloliquefaciens*, *Bacillus thuringiensis*, *Xenorhabdusbovienii*, *Pasteuria penetrans*, etc.) can be alternatively used for safer and cost-effective control of RKNs [34,35,36,37,38]. These biocontrol agents play crucial roles in controlling RKNs by producing various compounds in the rhizosphere which prevent the growth of RKNs by various mechanisms, like prevention of egg hatching, the damaging of second-stage juveniles and females, and the altering of sex ratio and feeding habits [34,36,37,39,40].

The present study was conducted to screen cultivars of eggplant so as to find the best and RKN-resistant candidate to act as a healthy rootstock for tomato scions. The eggplant cultivars were also screened using molecular markers to determine the resistance present in the eggplant cultivars. The resistant eggplant obtained in the screening experiment was used as the rootstock for the grafting of tomato scions. Finally, the grafted tomato plants were tested against RKN infection alone and with a biocontrol agent to enhance resistance against RKNs. Hence, this study provides integrated scientific approaches to curtailing the high losses caused by RKNs in tomato crops and offers a cost-effective and environmentally friendly solution to this devastating problem. We hope to extend this strategy to other crops for sustainable agriculture and pest management.

## 2. Materials and Methods

### 2.1. Root Knot Nematode Screening Experiment

#### 2.1.1. Root Knot Nematode Culture

The inoculum of *M. incognita* (family: Heteroderidae) for the screening of eggplant accessions/varieties was obtained from the Department of Nematology, College of Agriculture, CCSHAU (29.1427° N, 75.7040° E), Hisar, Haryana, India. The culture of *M. incognita* was multiplied and maintained in the Hisar Shymal (H8) brinjal variety. The eggs of *M. incognita* were obtained from diseased plants, and the inoculum was prepared as described by Hussey and Barker [41].

#### 2.1.2. Eggplant Varieties/Accessions and Potting Mixture

The seeds of thirty-five accessions/varieties of eggplant were collected from different sources (Table S1). The seeds of these accessions/varieties were sown in protrays containing a mixture of cocopeat, perlite, and vermiculite in the ratio of 3:1:1, respectively. The protrays were then kept in a greenhouse at 80–90% humidity and 28 °C temperature for seed germination. The potting mixture for RKN screening was prepared by using sandy loam soil and farmyard manure in the ratio of 3:1. It was steam-sterilized in an autoclave at 121 °C for 60 min before use. Thereafter, the potting mixture was allowed to cool and then used to fill earthen pots (1 kg soil capacity).

#### 2.1.3. Physical Screening of Eggplant Genotypes for Root Knot Nematode Infection

Thirty-day-old seedlings of the eggplant accessions/varieties were transferred into the earthen pots filled with steam-sterilized sandy loam soil and were infected with second-stage juveniles of *M. incognita* (inoculum 1 J2/gm of soil). The plants were grown using the standard package of practices and were screened for root knot nematode (RKN) infection. Observations were recorded after 40 days of inoculation to determine the All India Coordinated Research Project (AICRP) Root Knot Index (RKI). Based on the number of galls/root system and the number of eggs/root system, the AICRP Root Knot Index was determined, and screened plants were categorized as ‘Highly resistant’ (RKI-1): no galls; ‘resistant’ (RKI-2): 1–10 galls; ‘moderately resistant’ (RKI-3): 11–30 galls; ‘susceptible’ (RKI-4): 31–100 galls; and ‘highly susceptible’ (RKI-5): 101 and more galls.

#### 2.1.4. Number of Galls and Egg Masses

After 40 days of incubation, the roots of eggplants were carefully uprooted from the pots and were carefully washed to remove soil and manure without damaging the roots. After washing, the roots were thoroughly examined for the presence of root galls and egg masses. Bigger root galls were counted by eye, while small root galls and egg masses were counted using a simple microscope.

#### 2.1.5. Total Phenolic Content in Roots

The total phenolic content (TPC) in RKN-infected eggplant roots was determined by the Folin–Ciocalteu colorimetric method [42]. First, a 0.5 g eggplant root sample was finely ground using a pestle and mortar in 2 mL of distilled water and transferred to centrifuge tubes 5 mL in capacity. The mixture was then centrifuged at 8000 rpm for 10 min, and the supernatant was collected in a separate tube. The pellet was again ground using a pestle and mortar, and the extraction procedure was again repeated. Finally, both the supernatants were mixed, and the volume of the extracted mixture was made to 5 mL. For estimation of TPC, 1.5 mL of extract was placed in a tube, and 1.5 mL of distilled water was added to the extract to make the final volume 3 mL. Then, 0.5 mL of 1N Folin–Ciocalteu reagent (FCR) was added to this mixture. The solution was gently mixed and incubated at room temperature for 10 min. After incubation at room temperature, 2 mL of 7% Na_2_CO_3_ solution was added to the tube containing the extracted mixture and FCR. The content was gently mixed and incubated in boiling water for 1 min. After 1 min, the solution was taken out of the boiling water and cooled to room temperature for further investigation. Absorbance of the solution was measured at 650 nm in a colorimeter and equated with a blank. The TPC concentration (gallic acid equivalent) in the samples was determined as µg/g of root sample by equating with a standard curve.

### 2.2. Molecular Screening of Eggplant Varieties/Accessions

#### 2.2.1. DNA Isolation

Genomic DNA of thirty-five accessions/varieties was isolated from young leaves of individual eggplant accessions/varieties grown in a greenhouse using the CTAB extraction method [43,44,45]. The primers were synthesized by Sigma (St. Louis, MO, USA), and the DNA profile of thirty-five accessions/varieties was amplified using molecular markers (Table S2) for the presence or absence of a resistant band.

#### 2.2.2. PCR Amplification

DNA amplification was carried out in a Biorad T-100 programmable thermal cycler (BIORAD^TM^ INTERNATIONAL, Hercules, CA, USA) with a total volume of 25 μL of PCR reaction, consisting of 0.2 mM dNTPs, 1× PCR buffer, 1.5 mM MgCl_2_, 50–75 ng of DNA sample, 0.6 µM primer, and 1.5 U of *Taq* DNA polymerase. PCR amplification was performed with an initial denaturation step of 5 min at 94 °C, followed by 40 cycles of denaturation (94 °C, 1 min), annealing (54–60 °C, 45 s), and extension (72 °C, 30 s), followed by a final extension step at 72 °C for 7 min and a hold temperature of 4 °C. The amplified products were separated by gel electrophoresis and scored for the presence/absence of the alleles, as well as for the size of the allele.

#### 2.2.3. Grafting

In this experiment, the susceptible tomato cultivar was grafted on root knot nematode-resistant rootstocks of eggplant. The tomato cultivar Hisar Arun was used as the scion, while the resistant eggplant accession (BR3) obtained in the screening experiment was used as the rootstock. Various pieces of equipment, like surgical blades, grafting clips, supporting rods, and protrays, were used to carry out the grafting. The experiment was carried out in a greenhouse at 90–100% humidity and 25–28 °C temperature. Three different types of grafting (cleft, shaft, and shoot tip grafting) were carried out using resistant eggplant rootstocks (BR3) and tomato scions (Hisar Arun). The seedlings of tomato and eggplant were raised in the greenhouse in protrays containing a mixture of cocopeat, perlite, and vermiculite in the ratio of 3:1:1, respectively. Thirty-day-old seedlings were used for grafting. Seedlings of both tomato (scion) and eggplant (rootstock) were cut in compatible ends using sterile blades and were placed together. The scion and rootstock were held together with the help of a grafting clip and supporting rods. The experiment was conducted in a complete randomized design, and data were recorded for the number of days taken for successful grafting union and grafting success percentage. The best grafting technique was used for the production of an RKN-resistant tomato plant for further use in this study.

#### 2.2.4. Application of Biocontrol Agents to Enhance *Meloidogyne incognita* Resistance of Stably Grafted Tomato Plants

In this experiment, the biocontrol agents were used to further enhance the resistance against root knot nematode infection in grafted tomato plants. The grafted tomato plants were used as planting material, and *Trichoderma viride* (2 × 10^6^ cfu/gm), *Paecilomyces lilacinus* (2 × 10^6^ cfu/gm), and *Pseudomonas fluorescence* (2 × 10^8^ cfu/gm) were used as biocontrol agents. The grafted tomato plants were produced using shaft grafting in a greenhouse using resistant eggplant rootstocks (BR3) and Hisar Arun tomato scions. The grafted plants were kept in a greenhouse at 90–100% humidity and 25–28 °C temperature till successful grafting. The soil was prepared as described in the Screening Experiment Subsection. The pots filled with sterilized soil were kept in a greenhouse, and successfully grafted tomato plants were transferred to pots from protrays for application of biocontrol agents against RKNs. The experiment was conducted in a complete randomized design, and nine treatments were used to screen the grafted tomato plants against RKNs. The uninfected and untreated grafted tomato plants were used as the negative control (T1), while RKN (*M. incognita*)-infected and untreated grafted tomato plants were used as the positive control (T2). RKN (*M. incognita*)-infected grafted tomato plants treated with biocontrol agents *T. viride*, *P. lilacinus*, and *P. fluorescence* @2 g/kg of soil were denoted treatments T3, T4, and T5, respectively, while RKN (*M. incognita*)-infected grafted tomato plants treated with biocontrol agents *T. viride*, *P. lilacinus*, and *P. fluorescence* @4 g/kg of soil were denoted treatments T6, T7 and T8, respectively. Earthen pots filled with steam-sterilized sandy loam soil were incubated with 2 g/kg of soil and 4 g/kg of soil of biocontrol agents *T. viride*, *P. lilacinus*, and *P. fluorescense*. After seven days of incubation of the biocontrol agents, the soil was inoculated with 1 J2/gm of soil of RKNs (*M. incognita*). Finally, the successfully grafted tomato plants were transplanted into earthen pots. The pots were kept in the greenhouse, and grafted tomato plants were raised as per the standard package of practices. Observations were recorded after 40 days of transplanting, and data were recorded for the number of galls/root system, the number of eggs/root system, the final nematode population per 200 mL of soil, and total phenolic content. The data for plant height (cm), fresh weight of shoots (g), dry weight of shoots (g), fresh weight of roots (g), and dry weight of roots (g) were also recorded to study the effect of RKN infection on plant growth parameters.

#### 2.2.5. Estimation of RKN Population in 200 mL Soil Samples

The estimation of RKN population in 200 mL of soil was performed by using Cobb’s sieving and decanting technique [46] and Baermann’s funnel method [47]. The RKNs from soil samples were extracted, and the dilution method was used for counting the RKNs in the extracted samples. The extracted samples were incubated for 24 h, and the suspension was transferred to a counting dish by using a pipette [48]. The RKNs in the counting dish were counted under a stereo binocular microscope. Finally, the RKN population was estimated in 200 mL of soil.

#### 2.2.6. Experimental Design and Statistical Analysis

All experiments were performed in a complete randomized design with three replicates, and each experiment was repeated at least twice. The data were analyzed using OPSTAT software (beta version) available on CCSHAU, Hisar website for Analysis of Variance.

## 3. Results

### 3.1. Screening of Eggplant Varieties/Accessions Against RKNs

A total of thirty-five eggplant varieties/accessions were screened under controlled conditions against infection by RKNs, namely, *M. incognita*, and the data obtained during the screening experiment were used to categorize the eggplant varieties/accessions as susceptible or resistant. The results obtained during the study are presented in Table 1.

**Figure 1:**
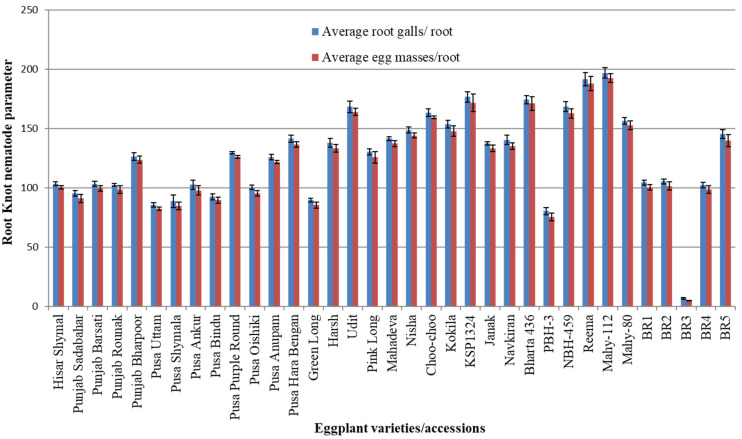
Screening of thirty-five eggplant varieties/accessions against RKN infection for root galls and egg masses: average root gall/root and average egg masses/root in different varieties/accessions of eggplant after 40 days of RKN infection.

**Figure 2:**
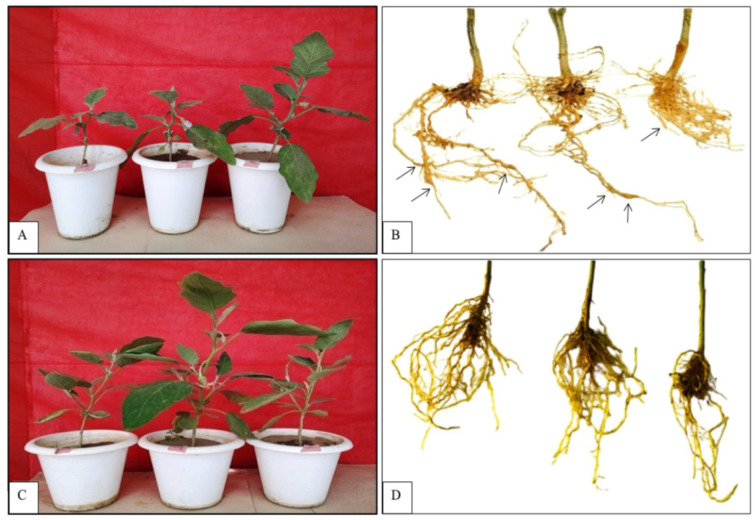
Screening of eggplant varieties/accessions against RKN infection (after 40 days of infection): (**A**) Most susceptible variety, Mahy-112. (**B**) Roots of variety Mahy-112 showing root galls and egg masses (black arrows showing prominent root galls). (**C**) Resistant accession BR3. (**D**) Roots of accession BR3 without root galls and egg masses.

Table 1 describes the result of the screening of thirty-five different varieties/accessions of eggplant against root knot nematode (*M. incognita*, inoculum 1 J2/gm of soil) infection. Seedling infestation data of eggplant accessions were analyzed according to the AICRP Root Knot Index. The average root galls and egg masses/root varied from 6.66 to 196.66 and from 4.66 to 192.66, respectively. The maximum root galls/root (196.66) and egg masses/root (192.66) were reported in the variety Mahy-112 (Figure 1 and Figure 2A,B), while the minimum root galls/root (6.66) and egg masses/root (4.66) were reported in accession BR3 (Figure 1 and Figure 2C,D). The AICRP Root Knot Index varied between 2.00 and 5.00. It was observed that none of the varieties/accessions screened in the present study were completely immune to root knot nematode infection. However, based on the AICRP Root Knot Index, one accession (BR3) was identified as resistant based on its low galling index and egg mass production; two varieties, Pusa Uttam and Pusa Bindu, were susceptible; and the remaining thirty-two varieties, i.e., Hisar Shymal, Punjab Sadabahar, Punjab Barsati, Punjab Rounak, Punjab Bharpoor, Pusa Shymala, Pusa Ankur, Pusa Purple Round, Pusa Oishiki, Pusa Anupam, Pusa Hara Bengan, Green Long, Harsh, Udit, Pink Long, Mahadeva, Nisha, Choo-choo, Kokila, KSP1324, Janak, Navkiran, Bharta 436, PBH-3, NBH-459, Reema, Mahy-112, Mahy-80, BR1, BR2, BR4, and BR5, were found to be highly susceptible to root knot nematode (*M. incognita*) infection.

### 3.2. Total Phenolic Content (TPC) in Roots

The total phenolic content in the roots of thirty-five varieties/accessions of eggplant screened against root knot nematode (*M. incognita*) infection was determined using the Folin–Ciocalteu colorimetric method [42]. The results obtained are presented in Table 2.

**Figure 3:**
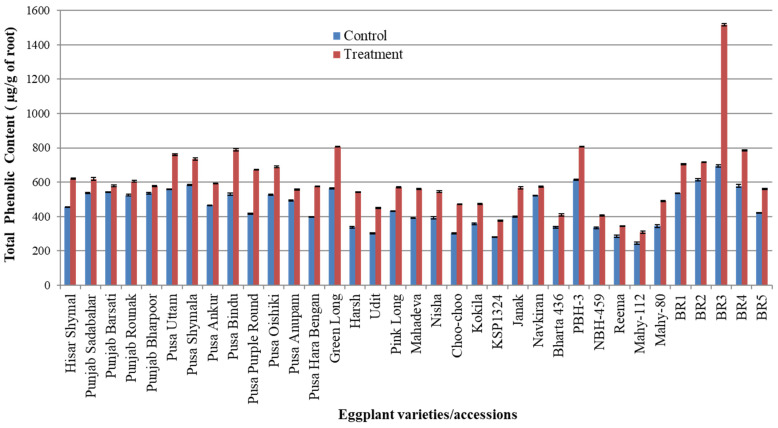
Total phenolic content/g root (equivalent to gallic acid) in thirty-five eggplant varieties/accessions of eggplant infected with RKN after 40 days of infection.

Table 2 describes the total phenolic content (TPC) of control and RKN-infected eggplant varieties/accessions. The maximum TPC (1515.92 µg/g of root) was observed in accession BR3, while the minimum TPC (309.25 µg/g of root) was observed in Mahy-112 roots infected with root knot nematodes (Figure 3). The phenolic content in plants is a good indicator of resistance reaction against invading pathogens. The increased TPC level in eggplant varieties/accessions showed a resistant reaction against root knot nematode infection. Hence, in terms of TPC production, a significant TPC increase (more than 2-fold) was observed in accession BR3 (1515.92 µg/g of root) compared with its control (694.44 µg/g of root). In the screening experiment, one accession (BR3) was identified as resistant based on its low galling index and egg mass production. Interestingly, this resistant accession also exhibited the highest total phenolic content (1515.92 μg/g) among all accessions and was used in a successive experiment for the grafting of tomato (Hisar Arun) scions.

The screening of germplasm is very important to determining resistance against RKNs. Resistant genotypes not only serve as good rootstocks for the grafting of susceptible scions but can also be used to develop new resistant varieties which are economically viable, thereby reducing the dependency on nematicides [49]. Eggplants are a good source of natural resistance against RKN infection and have previously been screened against root knot nematodes to determining their resistance properties [12,13,49,50,51,52]. In the present study, out of the thirty-five eggplant varieties/accessions screened, one accession, BR3, was found to be resistant against RKN infection, while two varieties, Pusa Uttam and Pusa Bindu, were susceptible, and the remaining thirty-two were highly susceptible to RKN infection. In a comparable study, Boiteux and Charchar [12] screened 39 eggplant varieties/genotypes in a greenhouse and found one genotype of eggplant to be resistant against RKN infection after 7 weeks of inoculation. They also confirmed the presence of the RKN-resistance properties in *Solanum torvum*. Similarly, Nayak and Sharma [50] screened 16 brinjal varieties and identified two brinjal varieties (Vijay and Annamalai) as resistant against RKNs. Dhivya et al. [53] screened eight Solanum rootstocks against RKNs under controlled conditions and observed that *S. torvum*, *Physalis peruviana*, and *S. sisymbrifolium* were found to be the most resistant rootstocks against RKNs, with the lowest galls/root, and also showed the highest phenolic content and enzymes related to defense from leaf samples. Similarly, Nayak and Pandey [54] identified 20 RKN-resistant varieties of brinjal out of 150 brinjal cultivars/varieties screened. Akhter and Khan [13] screened 30 brinjal varieties for resistance against RKNs, namely, *M. incognita* race-1. However, the result obtained by Akhter and Khan [13] is in contrast to our result. They reported variety Mahy-112 as tolerant and Mahy-80 as resistant against *M. incognita* race-1, while in the present study, it was observed that the varieties Mahy-112 and Mahy-80 were highly susceptible to RKN infection. Gawade et al. [49] have also identified 2 RKN (*M. incognita*)-resistant accessions out of 180 brinjal accessions screened. In a different study, Das et al. [55] screened twenty brinjal cultivars against RKNs (*M. javanica*) and found one cultivar (cv. Noagram) to be moderately resistant and the rest of the cultivars to be susceptible and highly susceptible. Similarly, in a recent study, El-Qurashi et al. [56] screened 11 eggplant cultivars from Saudi Arabia against RKNs (*M. javanica*) and found 1 cultivar to be highly resistant, 4 to be resistant, 2 to be moderately resistant, and 4 cultivars to be susceptible to RKNs (*M. javanica*). Hence, eggplant genotypes/cultivars provide a varying degree of resistance against RKN infection. However, the screening of eggplants against RKNs indicates that these plants may have some resistant properties/genes against infection by RKNs [12,13,14,15,56]. Resistant genotypes can be used to develop resistant varieties or can be used directly as rootstocks for the grafting of compatible scions to harness the natural resistance of genotypes without transferring a gene.

RKN infection induces characteristic gall formation on the roots of infected plants, with gall number determining infection severity [57]. The infection by RKNs is an interaction between the roots of the host plant and J2-stage RKNs which ultimately results in the formation of a feeding site and finally the swelling of roots [58]. Resistance of a host plant after RKN infection depends upon many factors, including hypersensitivity reaction and the release of phytoalexin and phenolic compounds [57]. Therefore, the phenolic compounds play an important role in the natural defense mechanism in host plants after RKN infection. In the present study, it was observed that the total phenolic content (TPC) of roots increased in all eggplant varieties/accessions after RKN infection. A more than two-fold increase in phenolic content was observed in resistant accession BR3 (1515.92 µg/g of root) compared with the control plant (694.44 µg/g of root). Nayak [59] similarly reported that RKN resistance correlates with phenolic accumulation, documenting 18.50%, 27.90%, and 42.90% increases in the infected eggplant varieties ‘Pusa Kranti’, ‘Kantabaigan’, and ‘Pusa Purple Long’, respectively, relative to controls. Shaaban et al. [60] analyzed the different chemicals in roots of infected eggplants using HPLC and found that the most tolerant variety (BPCL-1) showed the highest TPC (36,720.10 mAu) compared with the susceptible variety (108-3-1), where the TPC was the lowest (32,040.70 mAu) in infected roots. Accumulation of allelopathic compounds (phenols, phytoalexins, etc.) at the infection site of RKNs in the epidermis results in hypersensitivity reactions and necrosis of epidermal cells, which do not allow J2 RKNs to develop a feeding site, and finally, the RKNs die [57]. Gujjala et al. [61] also reported that TPC was the highest (67.05 mg/gm) in the resistant eggplant line (IIHR-824) upon infection by RKNs. These reports confirmed the direct role of these compounds in natural resistance exhibited by eggplant genotypes upon infection of by RKNs.

### 3.3. Molecular Screening of Eggplant Varieties/Accessions

Thirty-five eggplant varieties/accessions were screened using 12 molecular markers to study (Table S3) the presence of resistant loci at the molecular level. PCR amplification was performed, and the amplified products were separated by gel electrophoresis and scored for the presence/absence of the alleles, as well as for the size of the alleles. The amplified products of eggplant genotypes were studied for the presence of resistant or susceptible bands. Table S3 describes the amplification profile of thirty-five varieties/accessions using molecular markers. The twelve markers amplified the DNA of thirty-five varieties/accessions of eggplant, ranging from 50 bp to 2000 bp. However, the twelve markers used in the present study were unable to identify any resistant or susceptible band in the thirty-five varieties/accessions of eggplant (Figure S1).

In our study twelve molecular markers from different sources were used to screen thirty-five eggplant varieties/accessions [18,19,62,63,64,65,66,67,68]. The screening of eggplants against RKNs suggests that these plants may have some resistant genes [12,13,14,15]. One of the eggplant species, *Solanum torvum*, has been found to be extremely resistant against RKNs during physical screening, and a resistance gene highly similar to the RKN resistance gene has been identified in this species [16]. Ocal and Devran [69] also observed that *S. torvum* (Y28) was resistant to *M. incognita* populations under controlled conditions. A heat-stable gene, *SacMi*, has been reported in wild eggplant *S. aculeatissimum*, which is similar to the Mi genes and provides high resistance against RKNs [17]. The silencing of the *SacMi* gene using RNAi changes a resistant plant into a susceptible plant, indicating a role of this gene in RKN resistance in eggplant. In a study by Ainurrachmah et al., [70] screened the several eggplant accessions using Mi-1 gene markers (Rex and C8B) and found that these markers amplified DNA fragments of 550 bp in all eggplants, while in RKN-susceptible eggplants, only a 700 bp DNA band was amplified. RKN resistance conferred by the Mi gene has also been observed in eggplant and its wild relatives against important species of RKNs, i.e., M. arenaria, *M. incognita*, and *M. javanica* [19,62,63,64,65,66,67,68,70]. Wild plants screened using molecular markers can serve as a good source of genetic diversity, and RKN resistance genes from such wild sources can be transferred to susceptible cultivars by using interspecific breeding [17]. The 12 primers used in our study did not identify any resistant or susceptible band in the thirty-five eggplant accessions. Molecular markers can serve as excellent tools to identify specific loci linked to RKN resistance; however, their high cost, marker–character linkage issue, and reproducibility are major limitations in their application. Hence, the primers used in the present study did not identify any resistant or susceptible band in the eggplant accessions/varieties. However, one accession, BR3, was identified as resistant in the physical screening experiment against infection by RKNs.

### 3.4. Grafting of Tomato on RKN-Resistant Eggplant Rootstocks

**Figure 4:**
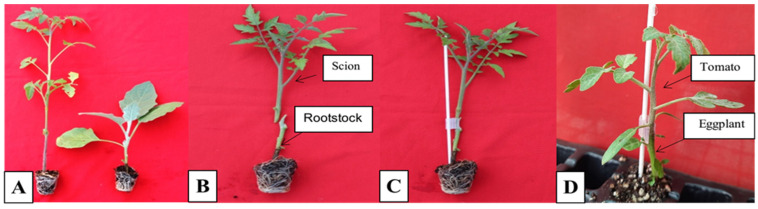
The shaft grafting of the Hisar Arun variety of tomato on eggplant (BR3) rootstock. (**A**) Tomato (Hisar Arun scion) and eggplant (BR3 rootstock) seedling. (**B**) Excised rootstock and scion (**C**) Insertion of scion into rootstock. (**D**) Successfully grafted plant of tomato (Hisar Arun) on eggplant BR3 rootstock.

In this experiment, scions of the tomato cultivar Hisar Arun was grafted on RKN-resistant eggplant BR3 rootstocks screened in experiments 1 and 2. Three different types of grafting (cleft, shaft, and shoot tip grafting) were used to standardize the best grafting technique (Table 3, Figure 4). Finally, the best grafting technique was used for the production of RKN-resistant tomato plants.

Out of three types of grafting techniques used in the present study, shaft grafting was found to be most effective for the grafting of tomato scions (Hisar Arun) on BR3 eggplant rootstocks with a 90% success rate. Cleft grafting, with 23.33% success, was the second best, while tip grafting, with a 13.33% success rate, was the least effective for the grafting of tomato scions (Hisar Arun) on BR3 eggplant rootstocks. In terms of the number of days required for successful grafting, shaft grafting was found to be most effective, which showed successful grafting in 21.27 days, followed by cleft grafting, which showed successful grafting in 29.44 days, while in tip grafting, successful grafting was observed in a maximum of 33.5 days. Hence, in the successive experiment, shaft grafting was used for grafting tomato cultivar Hisar Arun scions on root knot nematode-resistant BR3 rootstocks of eggplant. The successfully grafted tomato plants were used for a biocontrol screening experiment.

Grafting is a technique involving the forced joining of a rootstock and a scion of two living plants that ultimately grow as a single independent plant [28]. In the present study, three different types of grafting, i.e., cleft, shaft, and shoot tip grafting, were used for grafting tomato scions (Hisar Arun) on eggplant rootstocks (BR3). Shaft grafting with 90 per cent graft success was found to be the best amongst the three. In a similar study, Bahadur et al. [71] used splice or side grafting (shaft grafting) to produce grafted tomato plants using eggplant rootstocks. In a different study cleft grafting was found to be most effective for the grafting of tomato scions on wild *Solanaceae* rootstocks with 80–100% graft survival [25]. Our research study is also supported by the work by Black et al. [23], who reported that the plants from the same family tomato, such as *S. macrocarpan, S. aethiopicum*, and *S. torvum*, can be used as good rootstocks against Fusarium wilt, waterlogged conditions, drought tolerance, and root knot nematode infection. Eggplant rootstocks can be successfully used for the grafting of tomato, and the Asian Vegetable Research and Development Centre (AVRDC) specifically recommends the EG195 and EG203 eggplant accessions for the grafting of tomato to cope with bacterial wilt, Fusarium wilt, flooding, and RKNs [23]. The rootstocks used for the grafting of tomato may not only be resistant to biotic and abiotic stresses but also be compatible with the tomato scion for higher fruit production [28]. Resistant tomato hybrids are used as rootstocks in Europe for the grafting of tomato lines; however, in developing countries, their use is limited due to the higher cost of tomato hybrids. Hence, in developing countries, eggplants (*S. macrocarpan*, *S. aethiopicum*, *S. torvum*, etc.) have been used as rootstocks for the grafting of tomato lines [24]. Barrett et al. [30] conducted a two-year study to determine the cost-effectiveness of the grafting technique for root knot nematode (RKNs; *Meloidogyne* spp.) resistance and fruit yield in organic heirloom tomato and concluded that grafting might be a good choice for increasing the yield of tomato by overcoming severe RKN infection. Djidonou et al. [72] have also analyzed the production of grafted tomato plants in Florida (USA) and estimated the cost of grafted and non-grafted tomato plants at USD 0.65 and USD 0.15, respectively. Owusu et al. [31] conducted a study on the efficacy of grafted plants of tomato against RKNs. They used five tomato cultivars, among which ‘Jetsetter’, ‘Big Beef’, and ‘Celebrity’ were used as resistant rootstocks against RKNs and the cultivars ‘Power’ and ‘Tropimech’ were used as scions for grafting. They observed that the lowest nematode populations were in the cultivar ‘Power’ grafted on ‘Big Beef’, ‘Jetsetter’, and ‘Celebrity’ rootstocks, compared with non-grafted plants of this cultivar. The yield of grafted plants was also higher than that of non-grafted plants of the cultivar ‘Power’. In a study, Baidya et al. [32] grafted tomato on wild brinjal rootstocks (*S. sisymbrifolium*) and studied the efficacy of grafted plants against RKNs under field conditions. They observed that the roots of grafted tomato plants were free from root galls compared with non-grafted plants (which showed gall formation). The yield of grafted plants was also significantly higher (increased by 37%) than that of non-grafted plants. Draie [73] experimented on the grafting of susceptible tomato cultivars on broomrape resistant cultivars. Two broomrape-susceptible tomato cultivars, ‘Durinta’ and ‘Petula’, were grafted onto broomrape-tolerant rootstocks (‘Eldorado’) and broomrape-susceptible rootstocks (‘Maxifort’ and ‘Integro’) and were grown under conditions of infestation by branched broomrape in greenhouses. Vegetative and productive parameters were measured in each plant of cultivated tomato after four months of culture. The results showed that grafting improved the tolerance of the susceptible tomato cultivars to broomrape. Hence, the grafting of susceptible tomato cultivars onto tolerant/resistant rootstocks can be an important technique for tomato production under infestation conditions of broomrape disease.

### 3.5. Application of Biocontrol Agents to Enhance the Resistance Against RKNs

**Figure 5:**
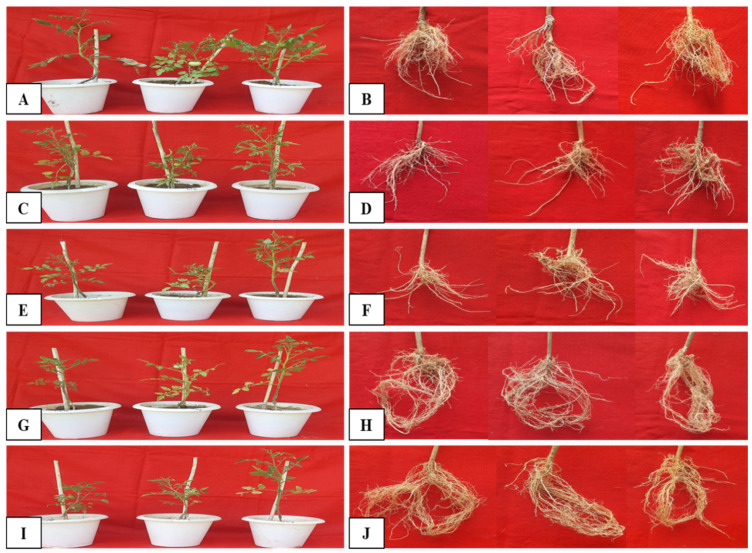
Testing of grafted tomato plants against RKNs with application of biocontrol agents. (**A**,**B**) Negative control plants (grafted tomato plants without root knot nematode infection and without biocontrol agents): (**A**) grafted tomato plants in pots; (**B**) roots of plants. (**C**,**D**) Positive control plants (grafted tomato plants with root knot nematode infection and without biocontrol agents): (**C**) grafted tomato plants in pots; (**D**) roots of plants. (**E**,**F**) Grafted tomato plants with root knot nematode infection and 4 g/kg of soil biocontrol agent *Trichoderma viride*: (**E**) grafted tomato plants in pots; (**F**) roots of plants. (**G**,**H**) Grafted tomato plants with root knot nematode infection and 4 g/kg of soil biocontrol agent *Paecilomyces lilacinus*: (**G**) grafted tomato plants in pots; (**H**) roots of plants. (**I**,**J**) Grafted tomato plants with root knot nematode infection and 4 g/kg of soil biocontrol agent *Pseudomonas fluorescens*: (**I**) grafted tomato plants in pots; (**J**) roots of plants.

The effect of three biocontrol agents (*T. viride*, *P. lilacinus*, and *P. fluorescence*) as soil amendment in pots on RKNs (*M. incognita*) infection and growth parameters of grafted tomato plants was studied. Table 4 describes the effect of 2 g/kg of soil and 4 g/kg of soil biocontrol agents on RKN (*M. incognita*) infection and growth parameters of grafted tomato plants on the 40th day of transplanting. It was observed that all three biocontrol agents effectively controlled the RKN infection. However, amongst the three biocontrol agents used, *P. lilacinus* @4 g/kg of soil (T7) was found to be the most effective (Table 4, Figure 5) in controlling RKN infection in grafted tomato plants, with the lowest number of root galls/root (1.33), egg masses (0.33), and final nematode population/200 mL of soil (40.66) compared with positive control plants (T2). On the other hand, grafted tomato plants infected with RKNs (positive control plants T2) showed the highest No. of root galls (8.66), egg masses (7.66), and final nematode population/200 mL of soil (227.33), followed by T3, with 7.66 root galls, 5.66 egg masses, and 175.0 final nematode population/200 mL of soil. It was observed that the use of *P. lilacinus* @4 g/kg of soil in grafted tomato plants reduced root galls, egg masses, and final nematode population by 84.65, 95.7, and 82.12 per cent, respectively, compared with the positive control plants (T2). In terms of plant growth parameters, the grafted tomato plants treated with *P. lilacinus* @4 g/kg of soil (T7) exhibited higher plant height (38.36 cm), fresh shoot weight (26.89 gm), dry weight of shoots (5.29 gm), fresh weight of roots (5.80 gm), dry weight of roots (1.91 gm), and total phenolic content (1691.80 µg/gm of root) compared with the positive control plants (T2). The results obtained in the present study clearly suggest that the use of RKN-resistant eggplant rootstock (BR3) for the grafting of susceptible tomato scions (Hisar Arun), along with the application of biocontrol agents, not only controlled infection by RKNs but also promoted the growth of the grafted tomato plants. Hence, the application of the biocontrol agent *P. lilacinus* can be used along with grafting to enhance protection against RKN infection in tomato.

RKNs are basically endoparasites which infect the host plant and create feeding sites and root galls [74]. The mechanism of host and RKN interaction is governed by a chemotactic mechanism where, upon injury, the roots release exudates, which are sensed by the RKN juvenile. This host plant interaction is the basis of RKN infection; hence, if some external nematicides are applied to prevent host and RKN interaction, then such nematicides can be used as a controlling agent for preventing infection by RKNs. Biocontrol agents such as fungi (*T. harzianum, T. viridae, P. lilacinus*, etc.) and bacteria (*P. fluorescens*, *B. amyloliquefaciens*, *B. thuringiensis*, *X. bovienii*, *P. penetrans*, etc.) have been alternatively used to control RKN infection [34,35,36,37,38]. These biocontrol agents play crucial roles in controlling RKNs by producing various compounds in the rhizosphere which prevent the growth of RKNs by various mechanisms, like prevention of egg hatching, the damaging of second-stage juveniles and females, and the altering of sex ratio and feeding habits [34,36,37,39,40]. Mokbel and Alharbi [75] effectively used various bacterial and fungal genera alone and in combination as biocontrol agents against RKN infection. They observed that the application of *P. fluorescens*, *Serratia marcescens*, *B. thuringiensis*, and *B. subtilis* significantly improved plant growth parameters, inhibited J2 activity by 50.5–90.3% and root galls and egg masses production by 56.5-86.8, and also reduced the population of J2/250 mL of soil. Similarly, *Saccharomyces cerevisiae*, *A. oligospora*, *Arthrobotrys conoides*, and *P. lilacinus* were also applied as biocontrol agents and significantly reduced J2/250 mL soil, egg masses, and root galls/root system (by 69.5–89.5%) and improved root and shoot dry weights of eggplant by 53.7–60.9%. Hence, it is evident from the above that bacteria and fungi can effectively control RKN infection, and these biocontrol agents are environmentally safe and cost-effective in comparison to chemical nematicides.

## 4. Conclusions

This study employed an integrated approach, screening eggplant accessions for RKN resistance, grafting, and biocontrol agent application, to develop RKN-resistant tomato plants. Resistant eggplant accessions serve as rootstocks for grafting or parental lines for hybrid resistant cultivars. Grafting harnesses rootstock resistance without genetic transformation. Combining biocontrol agents with grafting effectively controls RKN infection in tomato, reducing reliance on chemical nematicides while promoting environmental safety. This integrated strategy holds promise for application to other solanaceous crops facing RKN challenges.

## Figures and Tables

**Table 1:** Screening of thirty-five varieties/accessions of eggplant for root knot nematode infection and their categorization as susceptible or resistant based on AICRP Root Knot Index.

S. No.	Accessions/Varieties	Average Root Galls/Roots	Average Egg Masses/Roots	Average RKN Index	Categorization
1	Hisar Shymal	103.33 ± 1.66	100.33 ± 1.45	4.66 ± 0.33	HS
2	Punjab Sadabahar	95.33 ± 2.18	91.00 ± 3.51	4.66 ± 0.33	HS
3	Punjab Barsati	103.33 ± 2.18	99.33 ± 2.33	4.66 ± 0.33	HS
4	Punjab Rounak	102.33 ± 1.20	98.33 ± 3.28	4.66 ± 0.33	HS
5	Punjab Bharpoor	126.33 ± 3.18	123.66 ± 2.96	5.00 ± 0.00	HS
6	Pusa Uttam	85.66 ± 1.76	82.33 ± 1.33	4.00 ± 0.00	S
7	PusaShymala	88.66 ± 5.20	84.66 ± 3.18	4.33 ± 0.33	HS
8	Pusa Ankur	102.66 ± 3.93	97.66 ± 3.93	4.66 ± 0.33	HS
9	PusaBindu	92.33 ± 2.60	89.66 ± 2.60	4.00 ± 0.00	S
10	Pusa Purple Round	129.66 ± 0.88	126.00 ± 1.00	5.00 ± 0.00	HS
11	PusaOishiki	100.33 ± 1.85	95.33 ± 2.40	4.33 ± 0.33	HS
12	Pusa Anupam	125.66 ± 2.33	121.66 ± 1.20	4.66 ± 0.33	HS
13	Pusa Hara Bengan	141.33 ± 2.90	136.33 ± 2.33	5.00 ± 0.00	HS
14	Green Long	89.66 ± 1.66	85.33 ± 2.33	4.33 ± 0.33	HS
15	Harsh	137.66 ± 3.71	133.33 ± 3.33	5.00 ± 0.00	HS
16	Udit	168.33 ± 4.91	164.00 ± 3.05	5.00 ± 0.00	HS
17	Pink Long	130.33 ± 2.60	125.66 ± 4.84	5.00 ± 0.00	HS
18	Mahadeva	141.33 ± 1.85	137.33 ± 2.60	5.00 ± 0.00	HS
19	Nisha	148.66 ± 2.40	144.00 ± 2.00	5.00 ± 0.00	HS
20	Choo-choo	163.33 ± 3.33	159.33 ± 1.20	5.00 ± 0.00	HS
21	Kokila	153.66 ± 3.18	147.66 ± 4.33	5.00 ± 0.00	HS
22	KSP1324	176.66 ± 4.40	171.66 ± 7.26	5.00 ± 0.00	HS
23	Janak	137.33 ± 1.45	133.33 ± 2.84	5.00 ± 0.00	HS
24	Navkiran	140.33 ± 3.93	135.00 ± 2.64	5.00 ± 0.00	HS
25	Bharta 436	174.33 ± 3.48	171.00 ± 5.85	5.00 ± 0.00	HS
26	PBH-3	80.33 ± 2.90	75.33 ± 3.18	4.33 ± 0.33	HS
27	NBH-459	168.33 ± 4.05	162.66 ± 3.84	5.00 ± 0.00	HS
28	Reema	191.66 ± 5.54	187.66 ± 6.06	5.00 ± 0.00	HS
29	Mahy-112	196.66 ± 4.40	192.66 ± 3.66	5.00 ± 0.00	HS
30	Mahy-80	156.33 ± 2.90	152.66 ± 3.71	5.00 ± 0.00	HS
31	BR1	104.33 ± 2.18	100.33 ± 2.33	4.66 ± 0.33	HS
32	BR2	105.33 ± 2.18	101.33 ± 3.48	4.66 ± 0.33	HS
33	BR3	6.66 ± 0.66	4.66 ± 0.33	2.00 ± 0.00	R
34	BR4	102.33 ± 2.33	98.66 ± 3.28	4.33 ± 0.33	HS
35	BR5	145.33 ± 3.71	139.66 ± 5.23	5.00 ± 0.00	HS
Critical Difference (C.D.)		8.73	9.72	0.57	
Critical Variance (C.V.)		4.24	4.88	7.49	

Note: HS (highly susceptible); S (susceptible); R (resistant). Data: Means ± Standard errors; Critical Difference (C.D.) and Critical Variance (C.V.) at the significance level of 5% (α = 0.05).

**Table 2:** Total phenolic content (TPC g/root; equivalent to gallic acid) of control (uninfected) and infected plants of thirty-five varieties/accessions of eggplant on the 40th day of root knot nematode infection.

S. No.	Varieties/Accessions of Eggplant	TPC µg/g of Root in Uninfected Eggplant	TPC µg/g of Root in RKN-Infected Eggplant
1	Hisar Shymal	455.55 ± 2.22	621.47 ± 3.29
2	Punjab Sadabahar	537.77 ± 3.88	618.88 ± 8.91
3	Punjab Barsati	541.10 ± 1.78	578.51 ± 4.64
4	Punjab Rounak	524.44 ± 4.00	605.18 ± 5.60
5	Punjab Bharpoor	535.55 ± 4.89	575.55 ± 3.56
6	Pusa Uttam	558.33 ± 2.22	760.36 ± 4.27
7	PusaShymala	583.33 ± 2.22	735.92 ± 6.55
8	Pusa Ankur	464.99 ± 1.66	592.59 ± 2.67
9	Pusa Bindu	528.88± 6.66	787.77 ± 6.79
10	Pusa Purple Round	414.99 ± 2.77	671.84 ± 1.04
11	PusaOishiki	527.77 ± 3.33	688.88 ± 5.25
12	Pusa Anupam	492.77 ± 3.66	556.66 ± 2.31
13	Pusa Hara Bengan	397.77 ± 2.22	576.29 ± 2.25
14	Green Long	563.88 ± 3.66	806.66 ± 2.00
15	Harsh	337.22 ± 5.00	541.47 ± 2.09
16	Udit	302.77 ± 3.88	449.62 ± 2.26
17	Pink Long	431.10 ± 2.22	570.36 ± 1.83
18	Mahadeva	391.11 ± 1.11	559.99 ± 3.90
19	Nisha	392.77 ± 5.55	544.81 ± 3.76
20	Choo-choo	302.21 ± 3.33	472.96 ± 1.61
21	Kokila	357.77 ± 5.55	473.32 ± 2.31
22	KSP1324	280.55 ± 1.67	376.66 ± 3.33
23	Janak	399.44 ± 3.88	566.29 ± 5.74
24	Navkiran	522.22 ± 2.22	574.81 ± 3.29
25	Bharta 436	337.22 ± 5.00	408.88 ± 5.83
26	PBH-3	614.44 ± 3.33	806.66 ± 3.25
27	NBH-459	333.88 ± 5.00	405.55 ± 3.39
28	Reema	286.11 ± 6.11	343.70 ± 3.15
29	Mahy-112	244.44 ± 5.56	309.25 ± 5.73
30	Mahy-80	343.88 ± 8.33	491.10 ± 3.20
31	BR1	537.21 ± 0.58	704.81 ± 2.42
32	BR2	614.44 ± 6.67	714.81 ± 1.78
33	BR3	694.44 ± 5.56	1515.92 ± 5.74
34	BR4	577.77 ± 7.77	785.55 ± 4.13
35	BR5	421.66 ± 1.66	559.62 ± 4.17
CD		12.43	11.87
CV		1.34	1.19

Data: Means ± Standard errors; Critical Difference (C.D.) and Critical Variance (C.V.) at the significance level of 5% (α = 0.05).

**Table 3:** Success percentage of different types of grafting techniques used for the grafting of tomato (Hisar Arun) scions on eggplant (BR3) rootstocks.

S. No.	Type of Grafting	Success Percentage	Number of Days Taken for Grafting
1	Cleft Grafting	23.33 ± 3.33	29.44 ± 1.47
2	Shaft Grafting	90.00 ± 5.77	21.27 ± 0.19
3	Tip Grafting	13.33 ± 3.33	33.50 ± 0.86
	Critical Difference (C.D.)	15.18	3.50
	Critical Variance (C.V.)	17.65	6.12

Data: Means ± Standard errors; Critical Difference (C.D.) and Critical Variance (C.V.) at significance level of 5% (α = 0.05).

**Table 4:** Effects of biocontrol agents (@2 gm/kg of soil and 4 gm/kg of soil) on RKN infection and growth parameters of grafted plants of tomato (BR3 brinjal rootstocks and Hisar Arun scions) after 40 days of infection.

S. No.	Parameter Observed	* Negative Control (T1)	** Positive Control (T2)	Biocontrol Agents @2 g/kg of Soil			Biocontrol Agents @4 g/kg of Soil		
				*T. viride* (T3)	*P. lilacinus* (T3)	*P. fluorescence* (T4)	*T. viride* (T5)	*P. lilacinus* (T6)	*P. fluorescence* (T7)
1	Number of Root Galls/Root	0.00 ± 0.00	8.66 ± 0.33	7.66 ± 0.33	4.33 ± 0.33	4.66 ± 0.33	6.66 ± 0.66	1.33 ± 0.33	2.33 ± 0.33
2	Number of Egg Masses/Root	0.00 ± 0.00	7.66 ± 0.33	5.66 ± 0.66	2.66 ± 0.33	3.00 ± 0.57	3.33 ± 0.33	0.33 ± 0.33	1.33 ± 0.33
3	Final Nematode Population/200 mL of Soil	0.00 ± 0.00	227.33 ± 1.45	175.00 ± 2.88	86.33 ± 1.20	94.33 ± 1.76	105.33 ± 0.88	40.66 ± 2.18	71.00 ± 1.15
4	Plant Height (cm)	38.80 ± 0.26	34.33 ± 0.41	35.56 ± 0.41	36.36 ± 0.43	35.40 ± 0.32	36.10 ± 0.51	38.36 ± 0.51	35.43 ± 0.29
5	Fresh Weight of Shoots (g)	27.18 ± 0.43	24.52 ± 0.63	25.78 ± 0.23	26.30 ± 0.36	24.66 ± 0.12	25.40 ± 0.20	26.89 ± 0.31	26.02 ± 0.40
6	Dry Weight of Shoots (g)	5.26 ± 0.11	4.07 ± 0.06	4.86 ± 0.05	5.01 ± 0.10	4.69 ± 0.12	4.83 ± 0.08	5.29 ± 0.22	5.16 ± 0.10
7	Fresh Weight of Roots (g)	6.45 ± 0.05	7.79 ± 0.03	5.02 ± 0.06	5.50 ± 0.04	4.84 ± 0.05	4.71 ± 0.14	5.80 ± 0.04	5.25 ± 0.15
8	Dry Weight of Roots (g)	1.95 ± 0.02	1.26 ± 0.04	1.58 ± 0.08	1.87 ± 0.05	1.67 ± 0.03	1.82 ± 0.06	1.91 ± 0.08	1.75 ± 0.05
9	Total Phenolic Content µg/gm in Roots	670.73 ± 3.53	1493.21 ± 3.41	1519.16 ± 3.19	1588.96 ± 4.42	1580.53 ± 1.12	1572.26 ± 6.05	1691.80 ± 4.86	1604.43 ± 4.61
Critical Difference (C.D.)		3.36	3.63	4.17	4.38	2.08	5.81	5.09	4.51
Critical Variance (C.V.)		2.48	1.15	1.34	1.42	0.67	1.88	1.60	1.47

Data: Means ± Standard errors; Critical Difference (C.D.) and Critical Variance (C.V.) at the significance level of 5% (α = 0.05). * Negative control: uninfected and untreated grafted tomato plants; ** positive control: RKN-infected and untreated grafted tomato plants.

## Data Availability

The data presented in this study are available upon request from the corresponding author, as the data are related to the PhD thesis work of his student, and the data cannot be made public until the latter’s degree completion.

## Supplementary Materials

The following supporting information can be downloaded at: https://www.mdpi.com/article/10.3390/pathogens14121257/s1: Table S1: Different varieties/accessions of eggplant used for RKNs and molecular screening, Table S2: Different molecular markers used for the screening of thirty-five eggplant accessions, Table S3: Amplification profile of different molecular markers used for the screening of thirty-five eggplants rootstocks for identifying root knot nematode resistance bands, Figure S1: Amplification profile of thirty-five eggplants verities/accessions generated using primer TG263.
