# Supplementary material for: Grafting Tomato Scions on Root Knot Nematode (RKN)-Resistant Brinjal Rootstocks Complemented with Biocontrol Agents as an Integrated Nematode Management (INM) Strategy for the Development of RKN-Resistant Tomato
Source: Pathogens. 2025 Dec 8;14(12):1257. doi: 10.3390/pathogens14121257 (PMC12736213; doi:10.3390/pathogens14121257)
Supplement: Supplementary file 1 [file pathogens-14-01257-s001.zip › pathogens-3986412-supplementary.pdf]

Table S1: Different varieties/accessions of eggplant used for RKN and molecular screening.

S. No.	Name of the Variety/Accession	Source
1	Hisar Shymal (H8)	CCSHAU*
2	Punjab Sadabahar	PAU**
3	Punjab Barsati	PAU
4	Punjab Rounak	PAU
5	Punjab Bharpoor	PAU
6	Pusa Uttam	IARI***
7	Pusa Shymala	IARI
8	Pusa Ankur	IARI
9	Pusa Bindu	IARI
10	Pusa Purple Round	IARI
11	Pusa Oishiki	IARI
12	Pusa Anupam	IARI
13	Pusa Hara Bengan	IARI
14	Green Long	Shatabadi Seeds
15	Harsh	Shatabadi Seeds
16	Udit	Shatabadi Seeds
17	Pink Long	Shatabadi Seeds
18	Mahadeva	Clause Seeds
19	Nisha	Clause Seeds
20	Choo-choo	Clause Seeds
21	Kokila	Clause Seeds
22	KSP1324	Kalash Seeds
23	Janak	Kalash Seeds
24	Navkiran	Sungro Seeds
25	Bharta 436	East-West Seeds
26	PBH-3	Onkar Seeds
27	NBH-459	Nath Seeds
28	Reema	ACSEN Seeds
29	MAHY-112	Mahyco Seeds
30	MAHY-80	Mahyco Seeds
31	BR1	CCSHAU*
32	BR2	CCSHAU
33	BR3	CCSHAU
34	BR4	CCSHAU
35	BR5	CCSHAU

CCSHAU*- Chaudhary Charan Singh Haryana Agricultural University, Hisar; PAU**- Punjab Agricultural University, Ludhiana; IARI***- Indian Agricultural Research Institute, Delhi

Table S2: Different molecular markers used for screening of thirty-five eggplant accessions.

S. No.	Marker	Type of Marker	Marker sequence	Plants tested	Reference (s)
1	CT119	CAPS	F- TCAGGTATCGAACCAAAC<br>R- TAAAAGGTTCATCCTAATA	*Lycopersicon peruvianum* (*Solanum peruvianum*)	Ammiraju et al. (2003)
2	C8B	CAPS	F- TACCCACGCCCCATCAATG<br>R- TGCAAGAGGGTGAATATTGAGTGC		
3	Aps	CAPS	F-GGAACGTGGGTAGCATATGA<br>R- GCCAATGCTCATCAATGTGA		
4	C2S4	RAPD	CTAAGAGGAATCTCATCACAGG	*Lycopersicon esculentum* (*Solanum lycopersicum* L.)and *Lycopersicon peruvianum*(*Solanum peruvianum*)	Milligan et al. 1998
5	C1/2	RAPD	CAGTGAAGTGGAAGTGATGA		
6	Mi 1.2	Gene Specific	F-AAACCACTGTGGGTCCTCTGTT	*Solanum lycopersicum* L.	Patel et al. (2018)

			R- TGGATGATTGTATCATAAAGGGACAAATT		
7	REX	RAPD	F-TCGGAGCCTTGGTCTGAATT<br>R- GCCAGAGATGATTCGTGAGA	*Solanum lycopersicum* L.	Williamson et al. (1994)
8	Mi23	SCAR	F-TGG AAA AAT GTT GAA TTT CTT TTG<br>R- GCA TAC TAT ATG GCT TGT TTA CCC	*Solanum lycopersicum* L.	Seah et al. (2007)
9	Pmi	SCAR	F- GGTATGAGCATGCTTAATCAGAGCTCTC<br>R- CCTACAAGAAATTATTGTGCGTGTGAATG	*Solanum lycopersicum* L.	Arens et al. (2010)
10	TG-263	SCAR	F- GCTGAGAAATAAAGCTCTTGAGG<br>R- TACCCTTAATGCTTCGGCAGTGG	*Solanum lycopersicum* L.	Yaghoobi et al. (2005)
11	TG-180	SCAR	F- ATACTTCTTTGCAGGAACAGCTCAC<br>R- CACATTAGTGATCATAAAGTACCAG		
12	SAMI02	SacMi Gene Specific	F- CAGCGAAGTGGAAGTGATGA<br>R- GCAAAGTGAACCAAGTGCAA	*Solanum aculeatissimum*	Patent CN107287343A (2017)

Table S3: Amplification profile of different molecular markers used for screening of thirty-five eggplants rootstocks for identifying Root knot nematode resistance band.

S. No.	Marker	Type of Marker	Marker sequence	Bands desired for resistant/susceptible character	Bands amplified
1	CT119	CAPS	F- TCAGGTATCGAACCAAAC<br>R- TAAAAGGTTCATCCTAATA	450bp<br>Resistant: 280 bp, 170 bp<br>Susceptible: 250 bp, 170 bp<br>Heterozygous: 280 bp, 250bp, 170 bp	50bp
2	C8B	CAPS	F- TACCCACGCCCCATCAATG<br>R- TTGCAAGAGGGTGAATATTGAGTGC	Resistant: 400 bp, 360 bp<br>Susceptible: 360 bp	50 bp, 600 bp, 1300 bp
3	Aps	CAPS	F-GGAACGTGGGTAGCATATGA<br>R- GCCAATGCTCATCAATGTGA	Resistant: 1.0 kb, 0.6 kb<br>Susceptible: 1.6 kb	50 bp, 800 bp
4	C2S4	RAPD	CTAAGAGGAATCTCATCACAGG	Resistant: 1.6 kb	170 bp, 350 bp, 480bp, 800 bp, 1500 bp
5	C1/2	RAPD	CAGTGAAGTGGAAGTGATGA	Resistant: 1.6 kb	490 bp, 600 bp
6	Mi 1.2	Gene Specific	F- AAACCACTGTGGGTCCTCTGTT<br>R- TGGATGATTGTATCATAAAGGGACAAATT	Resistant: 347 bp	50 bp, 200 bp, 280 bp
7	REX	RAPD	F-TCGGAGCCTTGGTCTGAATT<br>R- GCCAGAGATGATTCGTGAGA	Resistant: 570 bp, 160 bp<br>Susceptible: 750 bp, 570 bp, 160 bp	50 bp, 120 bp, 180 bp, 600 bp, 1000 bp
8	Mi23	SCAR	F-TGG AAA AAT GTT GAA TTT CTT TTG<br>R- GCA TAC TAT ATG GCT TGT TTA CCC	Resistant: 455 bp or 380 bp<br>Susceptible: 420 bp	50 bp
9	Pmi	SCAR	F- GGTATGAGCATGCTTAATCAGAGCTCTC<br>R- CCTACAAGAAATTATTGTGCGTGTGAATG	Resistant: 550 bp<br>Susceptible: 350 bp	50 bp, 300 bp

10	TG-263	SCAR	F-GCTGAGAAATAAAGCTCTTGAGG<br>R-TACCCTTAATGCTTCGGCAGTGG	Resistant: 0.9 kb	50 bp, 400 bp, 580 bp, 800 bp, 1000 bp, 1200 bp
11	TG-180	SCAR	F-ATACTTCTTTGCAGGAACAGCTCAC<br>R-CACATTAGTGATCATAAAGTACCAG	Resistant: 1.2 kb	50 bp, 2000 bp
12	SAMI02	SacMi Gene Specific	F- CAGCGAAGTGGAAGTGATGA<br>R- GCAAAGTGAACCAAGTGCAA	Resistant: 1100 bp	300 bp, 580 bp, 800 bp, 1200 bp

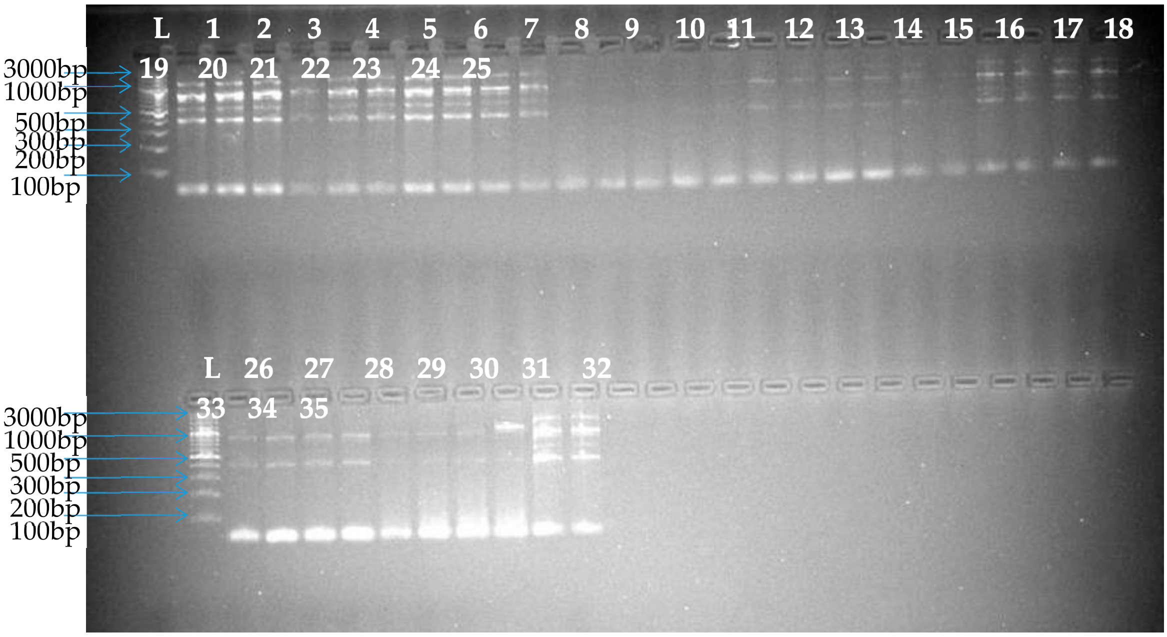

L-100bp ladder; 1-35 amplification profile of thirty five eggplant accessions/varieties

Figure S1: Amplification profile of thirty-five eggplants verities/accessions generated using primer TG263.
